# Clinical spectrum and predictors of severity of dengue among children in 2019 outbreak: a multicenter hospital-based study in Bangladesh

**DOI:** 10.1186/s12887-021-02947-y

**Published:** 2021-10-29

**Authors:** Md. Abdullah Saeed Khan, Abdullah Al Mosabbir, Enayetur Raheem, Ahsan Ahmed, Rashawan Raziur Rouf, Mahmudul Hasan, Fawzia Bente Alam, Nahida Hannan, Sabrina Yesmin, Robed Amin, Nazmul Ahsan, Sayeeda Anwar, Syeda Afroza, Mohammad Sorowar Hossain

**Affiliations:** 1 Department of Emerging and Neglected Diseases, Biomedical Research Foundation, Dhaka, Bangladesh; 2 Sir Salimullah Medical College and Mitford Hospital, Dhaka, Bangladesh; 3 Medicine Unit 1, BIRDEM, Dhaka, Bangladesh; 4 Department of Medicine, Dhaka Medical College & Hospital, Dhaka, Bangladesh; 5 Department of Medicine, Shaheed Suhrawardy Medical College & Hospital, Dhaka, Bangladesh; 6 Department of Paediatrics, Dhaka Medical College & Hospital, Dhaka, Bangladesh; 7 Department of Paediatrics, MH Samorita Hospital and Medical College, Dhaka, Bangladesh; 8 School of Environment and Life Sciences, Independent University, Dhaka, Bangladesh

**Keywords:** Dengue, Children, Pediatric, Severity predictors, 2019 outbreak, Dhaka, Bangladesh

## Abstract

**Background:**

The mosquito-borne arboviral disease dengue has become a global public health concern. However, very few studies have reported atypical clinical features of dengue among children. Because an understanding of various spectrums of presentation of dengue is necessary for timely diagnosis and management, we aimed to document the typical and atypical clinical features along with predictors of severity among children with dengue during the largest outbreak in Bangladesh in 2019.

**Methods:**

We conducted a cross-sectional study between August 15 and September 30, 2019. in eight tertiary level hospitals in Dhaka city. Children (aged < 15 years) with serologically confirmed dengue were conveniently selected for data collection through a structured questionnaire. Descriptive, inferential statistics, and multivariable logistic regression were used to analyze data.

**Results:**

Among the 190 children (mean age 8.8 years, and male-female ratio 1.22:1) included in the analysis, respectively 71.1 and 28.9% children had non-severe and severe dengue. All children had fever with an average temperature of 103.3 ± 1.2 F (SD). Gastrointestinal symptoms were the most common associated feature, including mostly vomiting (80.4%), decreased appetite (79.5%), constipation (72.7%), and abdominal pain (64.9%). Mouth sore, a less reported feature besides constipation, was present in 28.3% of children. Atypical clinical features were mostly neurological, with confusion (21.3%) being the predominant symptom. Frequent laboratory abnormalities were thrombocytopenia (87.2%), leucopenia (40.4%), and increased hematocrit (13.4%). Age (AOR 0.86, 95%CI 0.75–0.98, *p* = 0.023), mouth sore (AOR 2.69, 95%CI 1.06–6.96, *p* = 0.038) and a decreased platelet count (< 50,000/mm^3^) with increased hematocrit (> 20%) (AOR 4.94, 95%CI 1.48–17.31, *p* = 0.01) were significant predictors of severity.

**Conclusions:**

Dengue in children was characterized by a high severity, predominance of gastrointestinal symptoms, and atypical neurological presentations. Younger age, mouth sores, and a decreased platelet with increased hematocrit were significant predictors of severity. Our findings would contribute to the clinical management of dengue in children.

**Supplementary Information:**

The online version contains supplementary material available at 10.1186/s12887-021-02947-y.

## Background

Dengue has become a global public health concern, especially in most tropical and subtropical countries. It is a mosquito-borne (*Aedes aegypti and Aedes albopictus)* arboviral disease caused by the dengue virus (DENV) [1]. Over the last 60 years, the dengue virus has spread to over 130 countries, causing nearly 10,000 deaths and 100 million symptomatic cases every year [2, 3]. Besides, more than 50% of the global population are at risk of dengue transmission, with the vast majority in Asia, followed by Africa and America [4]. It is one of the leading causes of death among children in Southeast Asia [2].

In Bangladesh, the first recognized dengue outbreak was reported in the capital city, Dhaka in 1964 [5]. Subsequently, sporadic dengue cases were reported [6] until 2000, when the first major epidemic occurred throughout the major cities and towns of Bangladesh [7]. In 2019 the most extensive and deadliest outbreak of dengue occurred in the history of Bangladesh. Over 100,000 people were hospitalized, and 129 deaths were recorded [8]. The unofficial number of cases and deaths might be higher as the health reporting system is poor in the country. The high mortality was suspected to be associated with a high incidence of dengue shock syndrome (DSS) and secondary dengue infections [9].

DENV has four different serotypes (DENV-1 to 4). A peculiarity of DENV infection is that infection by one serotype produces serotype-specific lifelong immunity; however, contrary to giving protection or remaining neutral against other serotypes, a secondary infection by a heterogeneous serotype often results in severe disease by a mechanism called antibody dependent enhancement (ADE) [10]. All four serotypes have been isolated in Bangladesh with a predominance of DENV-3 till 2002 [11, 12]. After 2002, DENV-1 and DENV-2 were the prevalent serotypes, which increased the susceptibility of severe secondary infection by other serotypes [13]. In 2017, the DENV-3 serotype reemerged, causing a sharp rise of dengue cases in 2018 [14]. Thereafter, the 2019 outbreak was predominantly caused by type I genotype of the DENV-3 serotype [15].

Among children, nearly 95% of dengue cases are aged less than 15 years [16]. Owing to their immature hemodynamic system, children and particularly infants, tend to develop severe dengue disease [17, 18]. National surveillance data from Asian countries show that infants under 1 year of age and children aged 4–9 have consistently been at the highest risk for severe dengue disease [18].

Being a systemic disease, the clinical features of dengue show wide-ranging variations. Many of the clinical features were explored, but some (like constipation, mouth sores) are seldom reported. Moreover, children with dengue show significant variations in symptoms and signs in comparison to adults. Pain symptoms of dengue infection are less common among children [19–21]. Severe infection, when present, maybe preceded by so-called ‘warning’ signs [22]. Many clinical and laboratory factors have been attributed to the severity of the disease [23]. Early detection and appropriate management of severe dengue can reduce dengue-associated mortality [24]. Hence characterization of typical and atypical clinical features and determinants of severity, especially in the context of an outbreak, is essential. Previous studies have described the clinical presentations of dengue in the pediatric population of Bangladesh [25–28] from single centers and with a small number of samples. Globally, detailed profiling of clinical features, including usual and unusual clinical presentations of dengue in the pediatric population, is lacking. We hypothesized that the ongoing outbreak was characterized by increased severity and atypical clinical features suggestive of multi-system involvement among children with dengue. This study aimed to investigate that hypothesis, describe the typical and atypical clinical features, and model the clinical predictors of the severity of dengue among children in the setting of Bangladesh.

## Methodology

### Case definition

In Bangladesh, the National Guideline for Clinical Management of Dengue was published in collaboration with the World Health Organization (WHO) in 2018 for efficient management of dengue cases in the context of the country [29]. Based on this guideline, symptomatic dengue cases were initially classified into three major categories based on their severity: dengue fever (group A), and dengue fever with warning signs (group B), and severe dengue (group C). Here the former two (group A and B) are non-severe groups. A detailed description of the defining features is listed in Table S1 (Additional file 1). We also used the classical WHO categories of dengue, namely, dengue fever (DF), dengue hemorrhagic fever (DHF), dengue shock syndrome (DSS), and expanded dengue syndrome (EDS) for comparison purposes. EDS is an entity that was initially absent in the dengue management guideline published by WHO in 2009 [22] but was later incorporated in the revised and expanded edition of the guideline in 2011 [30]. This term was coined by WHO to describe cases that do not squarely fall within the definition of DHF and DSS and is characterized by atypical findings of dengue [31].

### Participants, study design, and data collection

This cross-sectional study was conducted during the peak of the 2019 dengue outbreak from August 15 to September 30, 2019. The study included hospitalized children with laboratory-confirmed dengue and was carried out in accordance with the Declaration of Helsinki. Children were defined as patients with age 14 years or less. Diagnosis was confirmed serologically by positive for the dengue nonstructural glycoprotein 1 (NS1) and/or dengue IgM antibodies. A total of 278 hospitalized children with dengue symptoms were enrolled; of which 237 children were serologically confirmed. After exclusion of incomplete data (19.8%), 190 cases of children with dengue were analyzed. Patients were recruited from five government hospitals and three private hospitals in the capital city of Bangladesh. The data collection centers with the number of pediatric patient data initially collected from each center (given in parenthesis) are as follows. Dhaka Medical College Hospital (*n* = 95), Kurmitola General Hospital (*n* = 49), Mugda Medical College Hospital (*n* = 37), Sir Salimullah Medical College Hospital (*n* = 1), Suhrawardy Medical College Hospital (*n* = 11), Popular Medical College Hospital (*n* = 17), Dr. Sirajul Islam Medical College Hospital (*n* = 16), and MH Samaarita Medical College Hospital (*n* = 10) (Figure S1) (Additional file 1). The initiative was taken by the Biomedical Research Foundation, Bangladesh. A team of 85 volunteer researchers composed of undergraduate and graduate medical students, clinicians, public health professionals, and statisticians got involved in this study.

Data was collected using a structured questionnaire via face-to-face interview by the trained volunteer medical students and medical doctors during the convalescent phase of the disease. The questionnaire was developed based on previously published literature and discussion with a multidisciplinary team, including clinicians. The questionnaire was pretested in a sample of 20 patients and any inter-observer variation was resolved through discussion among the team members. Presenting symptoms were grouped according to the system involved. We also incorporated a list of unusual symptoms in the questionnaire, based on the atypical manifestations of dengue [29, 32], to draw a picture of atypical presentations during the outbreak (see Additional file 2).

Enumerators were given one-day training on the use of the questionnaire. Guardians/parents of the respondents were asked about the demographic profile and clinical presentation during their first week of presentation where necessary. The investigation profile was collected from their hospital records. For hematological investigations, only the highest or lowest value of a test was collected when more than one record was found.

### Statistical analysis

Descriptive and inferential statistical procedures were used to illustrate the clinical profile of DENV infection and to ascertain the factors associated with severe infection. Descriptive statistics were reported as percentage and means when applicable along with standard deviation. Missing cases were excluded from bivariate analysis and data were expressed among the available responses for different variables. Completed data collection forms were scrutinized by data collection supervisors. Verified data were entered and subsequently managed using REDCap electronic data capture tool hosted at BRF server [33]. Data were analyzed using R statistical software. For testing association between categorical data, Pearson’s chi-square test was used, and Yate’s correction for continuity was applied where appropriate. We performed an independent sample t-test when comparing the means of continuous variables. Logistic regression analysis was used to see associations. A two-tailed *p*-value smaller than 0.05 was considered statistically significant.

### Ethical consideration

The study was approved by the Ethical Review Committee (ERC) of Biomedical Research Foundation (Memo no:BRF/ERB/2019/017). Written approval was taken from the authority of the selected hospitals before data collection. As approved by ERC, verbal and/or written informed consent was obtained from guardians of each participant as per their convenience. Trained enumerators first approached and explained consent forms to the prospective participants, and the study questionnaire was shared or discussed with them. After obtaining consent (oral and/or written) from the guardians or parents of the participants, they were registered for face-to-face interview and hospital document review. Some of them could not sign their names, in which case the questionnaire was marked indicating a case with verbal consent.

## Result

### Characteristics of the children

Out of 190 pediatric dengue patients, 179 (94.2%) cases were positive for NS1 antigen, and 11 (5.8%) cases for IgM antibody. Overall, children with dengue had an average age of 8.8 ± 3.7 years with a slight male predominance (1.22:1). However, severe cases showed slightly male predominance. The majority (46.1%) of the children belonged to the age group of 10–14 years. According to severity, about 20% fell under group A, 51% group B, and 29% group C. Therefore, more than two-third (71.1%) of the children were non-severe. According to the WHO dengue classification by symptoms, over two-thirds (63.7%) of children presented with DF while 10, 23.2, and 3.2% had DHF, DSS, and EDS, respectively. Past history of dengue and chikungunya were present only in 1.3 and 7.4% of children respectively. The mean age of severe (7.4 years) dengue cases was significantly lower than that of non-severe (9.4 years) dengue cases (*p* < 0.001). Other socio-demographic parameters were comparable between severe and non-severe groups (Table 1).

**Table 1:** Characteristics of the patients

Variable	Categories	Non-severe n (%)	Severe n (%)	p	Total n (%)
Total N (%)		135 (71.1)	55 (28.9)		190 (100)
**Age in years**	Mean (SD)	9.4 ± 3.8	7.4 ± 3.1	< 0.001	8.8 ± 3.7
**Age group**	< 5	16 (11.9)	11 (20.0)		27 (14.2)
	5–9	48 (35.6)	29 (52.7)		77 (40.5)
	10–14	71 (52.6)	15 (27.3)		86 (45.3)
**Sex**	Female	58 (43.0)	28 (51.9)	0.268	86 (45.5)
	Male	77 (57.0)	26 (48.1)		103 (54.5)
**Monthly Family Income (BDT)**	< 15,000	37 (30.8)	21 (40.4)	0.282	58 (33.7)
	15,000- < 25,000	49 (40.8)	21 (40.4)		70 (40.7)
	25,000- < 50,000	25 (20.8)	9 (17.3)		34 (19.8)
	50,000- < 100,000	8 (6.7)	0		8 (4.7)
	> 100,000	1 (0.8)	1 (1.9)		2 (1.2)
Residence	Flat	81 (60.9)	26 (49.1)	0.355	107 (57.5)
	Tin shade	41 (30.8)	24 (45.3)		65 (34.9)
	Single storied house	8 (6.0)	3 (5.7)		11 (5.9)
	Slum	2 (1.5)	0		2 (1.1)
	Other	1 (0.8)	0		1 (0.5)
Blood group	A+	13 (16.0)	7 (18.9)	0.896	20 (16.9)
	AB-	1 (1.2)	0		1 (0.8)
	AB+	10 (12.3)	5 (13.5)		15 (12.7)
	B-	4 (4.9)	1 (2.7)		5 (4.2)
	B+	18 (22.2)	11 (29.7)		29 (24.6)
	O-	1 (1.2)	0		1 (0.8)
	O+	34 (42.0)	13 (35.1)		47 (39.8)
History of Dengue/ Chikungunya	1: Dengue	2 (1.7)	1 (1.4)	0.450	3 (1.6)
	2: Chikungunya	12 (8.9)	5 (3.6)		14 (7.4)
	Neither	121 (89.6)	52 (94.5)		173 (91.1)
Classification by severity	Group A (Dengue)	39 (28.9)	0	< 0.001	39 (20.5)
	Group B (Dengue with warning signs)	96 (71.1)	0		96 (50.5)
	Group C (Severe dengue)	0	55 (100.0)		55 (28.9)
Classification by symptom	DF	121 (89.6)	0	< 0.001	121 (63.7)
	DHF	12 (8.9)	7 (12.7)		19 (10.0)
	DSS	1 (0.7)	43 (78.2)		44 (23.2)
	EDS	1 (0.7)	5 (9.1)		6 (3.2)

Data is presented as mean ± SD and n (%) as appropriate; Percentage is expressed among available responses (after excluding missing values)

*p* value determined by chi-square test and independent samples t test as appropriate

### Clinical manifestations of the children

#### Symptoms

Table 2 enlists the frequently occurring general, systematic and unusual clinical presentation of children in relation to severity. Additional less frequent freatures are described in Table S2 (Additional file 1). All of the children presented with fever. The average maximum recorded temperature was 103.3 F (SD ± 1.2). The average duration of fever was 5.4 days (SD ± 2.8). However, it was significantly higher in patients with severe disease than non-severe (6.2 ± 4.1 vs. 5.1 ± 2.0 days, p0.043). Other common presenting symptoms were mostly gastrointestinal, including vomiting (80.4%), lethargy (78.3%) decreased appetite (79.5%), constipation (72.7%), and abdominal pain (64.9%). Itching (33.2%), mouth sore (28.3%), and skin rash (27.7%) were common muco-cutaneous manifestations. Arthralgia was present in 40.8% of patients. About 24% of children experienced hemorrhagic manifestations. Epistaxis (6.3%) and malena (5.8%) were the most frequent bleeding manifestations, followed by subconjunctival hemorrhage (4.2%) and gum bleeding (3.7%). Most of the symptoms were similar between patients with non-severe and severe disease. Mouth sores and malena were significantly associated with severe disease. Interestingly, hair loss was present in 9 (6.9%) patients with the non-severe disease, while no patients with severe disease experienced it.

**Table 2:** General and systemic clinical manifestations of patients

Variable	Non-severe n (%)	Severe n (%)	p	Total n (%)
**Total**	**135 (71.1)**	**55 (28.9)**		**190 (100%)**
***General manifestations***				
**Fever and associated features**				
Fever	135 (100)	55 (100)		190 (100%)
Highest recorded temperature (F)	103.3 ± 1.2	103.3 ± 1.3	0.895	103.3 ± 1.2
Duration of fever (days)	5.1 ± 2.0	6.2 ± 4.1	**0.043**	5.4 ± 2.8
Lethargy	101 (79.5)	40 (75.5)	0.547	141 (78.3)
Headache	91 (69.5)	35 (63.6)	0.438	126 (67.7)
Backache	52 (40.3)	14 (27.5)	0.107	66 (36.7)
Retroorbital pain	44 (33.8)	19 (35.8)	0.796	63 (34.4)
**Muco-cutaneous manifestations**				
Itching	44 (33.3)	18 (32.7)	0.936	62 (33.2)
Mouth sores	31 (23.5)	22 (40.0)	0.022	53 (28.3)
Rash	34 (25.4)	18 (33.3)	0.270	52 (27.7)
**Joint manifestations**				
Arthralgia	54 (41.2)	21 (39.6)	0.842	75 (40.8)
***Danger ‘signs’***				
**Gastrointestinal features**				
Vomiting	107 (79.9)	45 (81.8)	0.757	152 (80.4)
Decreased appetite	109 (80.7)	42 (76.4)	0.498	151 (79.5)
Constipation	36 (72.5)	38 (73.1)	0.939	133 (72.7)
Abdominal Pain	86 (64.7)	36 (65.5)	0.917	122 (64.9)
Loose motion	61 (45.2)	20 (37.0)	0.307	81 (42.9)
**Hemorrhagic manifestation**				
Any Hemorrhage	23 (18.4)	19 (36.5)	0.010	42 (23.7)
Nasal bleeding (Epistaxis)	8 (5.9)	4 (7.3)	0.729	12 (6.3)
Malena	3 (2.2)	8 (14.5)	0.001	11 (5.8)
***Unusual manifestations***				
**Neurological symptoms**				
Confusion	28 (21.1)	12 (22.2)	0.860	40 (21.4)
Blurring of vision	16 (12.0)	12 (21.8)	0.086	28 (14.9)
**Others**				
Palpitation	11 (8.4)	11 (20.4)	0.022	22 (11.9)
***Signs***				
Anemia/Pallor	18 (14.3)	9 (17.0)	0.646	27 (15.1)
Dehydration	13 (10.4)	8 (15.7)	0.326	21 (11.9)
**Features of plasma leakage and shock**				
Clinical accumulation of fluid	12 (9.3)	17 (31.5)	< 0.001	29 (15.8)
Cold clammy skin	48 (37.2)	39 (72.2)	< 0.001	87 (47.5)
Excessive sweating	52 (40.0)	23 (42.6)	0.745	75 (40.8)
Oliguria/Anuria	27 (20.8)	21 (39.6)	0.009	48 (26.2)
Dyspnea	17 (12.8)	14 (25.9)	0.028	31 (16.6)
Loss of/impaired consciousness	3 (2.4)	11 (20.4)	< 0.001	14 (7.7)

Data is presented as mean ± SD and n (%) as appropriate; Percentage is expressed among available responses (after excluding missing values)

*p* value determined by chi-square test and independent samples t test as appropriate

#### Atypical symptoms

Several atypical manifestations were noted among patients with most frequent being confusion (21.4%), and blurring of vision (14.9%) (Table 2). Additionally, palpitation (11.9%), chest tightness (9.8%), sensory impairment (9.7%), convulsion (7.0%), disorientation (5.9%), neck stiffness (5.9%), palpable lymph nodes (3.1%), auditory hallucination (2.7%), transient amnesia (1.6%), altered mental state (1.6%) and facial deviation (0.5%) occurred infrequently among participants (Table S2). All the atypical manifestations were comparable between non-severe and severe groups except convulsion and palpitation, which were significantly higher among patients with severe disease.

#### Signs

Among features of plasma leakage and shock, cold-clammy skin (47.5%) and excessive sweating (40.8%) were most prominent. Moreover, 15.8% of children had obvious manifestations of clinical fluid accumulation (Table 2). Ascites, pleural effusion, and anasarca were present in 6.8, 5.8 and 2.6% patients respectively. While anemia and dehydration were found in 15.1 and 11.9% of children, respectively. Only three children had hepatomegaly (1.8%) (Table S2).

#### Laboratory investigations

Thrombocytopenia (< 150,000/mm^3^) was present in most children (87.2%), leucopenia (< 4000/mm^3^)in 40.4% and increased hematocrit (> 20% from baseline) in 13.4% of children (Table 3).

**Table 3:** Investigation profile of patients (*n* = 190)

Variable	Non-severe n (%)	Severe n (%)	p	Total n (%)
**Hematological profile**				
Increased hematocrit (> 20% from baseline)	13 (9.8)	12 (22.6)	0.157	25 (13.4)
Reduced hemoglobin level (< age adjusted minimum)	53 (39.6)	19 (35.2)	0.577	72 (38.3)
Thrombocytopenia (< 150,000/ mm^3^)	112 (84.2)	52 (94.5)	0.053	164 (87.2)
Leucopenia (< 4000/ mm^3^)	53 (42.4)	19 (35.8)	0.415	72 (40.4)

*p* value determined by chi-square test

#### Variations in clinical presentations by gender and age

Variation in clinical presentation was noted across gender (Table S3) (Additional file 1). Retro-orbital pain, backache, itching, hair loss, arthralgia, and leucopenia were significantly more common among girls than boys. Auditory hallucination was found only among boys (*n* = 5, *p* = 0.038). Other features showed statistically similar distribution.

Age-related variations among some clinical features were also documented (Table S4) (Additional file 1). Headache and loose motion were significantly more common among older groups (10–14 years) of children than other groups. Backache was significantly higher in the older (10–14) group compared to younger ones. Rash, decreased appetite, and constipation was significantly higher in the youngest group (< 5 years). Mouth sores were significantly more frequent in children < 10 years. Vomiting, hemorrhage and cold clammy skin was present in significantly higher proportion in the middle group (5–9 years) than other groups. Proportion of cases with severe dengue and dengue with warning signs were higher in respectively < 5 years and 5–9 years in comparison to the rest of the children.

### Determinants of severity among children

Factors which differed among children in relation to age, sex and severity were included in a predictive model to determine their predictive values for severe dengue among children (Table 4). These included child’s age, sex, certain clinical features (backache, retroorbital pain, oral ulcer, rash, arthralgia, vomiting, abdominal pain, loose motion, and constipation) and investigation (decreased platelet < 150,000/mm^3^ with increased hematocrit > 20% and leucopenia). Severity defining features like hemorrhage and signs of plasma leakage and shock were excluded from the model to avoid incorporation bias. Several atypical features were excluded due to their low count. Abdominal pain was kept in the model despite showing non-significant variation across groups as severe abdominal pain is a warning sign. In the final model age, oral ulcer and decreased platelet with increased hematocrit count was found to be a significant predictor of severity among children. One-year increase in age was associated with 14% reduction in risk of severity (OR 0.86, 95%CI 0.75–0.98, *p* = 0.023). Presence of mouth sores was associated with significantly higher odds of developing severe disease (OR 2.69, 95% CI 1.06–6.96, *p* = 0.038). A decreased platelet count with increased hematocrit was associated with 4.94 times higher odds (95%CI 1.48–17.31, *p* = 0.01) of developing severe dengue.

**Table 4:** Logistic regression analysis of factors predicting severe dengue among children

Variables	Unadjusted OR (95%CI)	***P*** value	Adjusted OR (95%CI)	***P*** value
**Age in years**	**0.86 (0.79–0.94)**	**0.001**	**0.86 (0.75–0.98)**	**0.023**
Gender (Female vs Male)	1.43 (0.76–2.70)	0.269	2.02 (0.78–5.35)	0.150
Backache	0.56 (0.27–1.12)	0.109	0.41 (0.13–0.19)	0.113
Retro orbital pain	1.09 (0.55–2.12)	0.796	1.53 (0.52–4.62)	0.441
**Mouth sores**	**2.17 (1.10–4.26)**	**0.024**	**2.69 (1.06–6.96)**	**0.038**
Rash	1.47 (0.73–2.91)	0.271	1.30 (0.51–3.21)	0.572
Arthralgia	0.94 (0.48–1.79)	0.842	0.46 (0.16–1.24)	0.136
Vomiting	1.14 (0.52–2.64)	0.757	1.17 (0.37–4.00)	0.789
Abdominal pain	1.04 (0.54–2.03)	0.917	0.74 (0.28–1.94)	0.540
Loose motion	0.71 (0.37–1.36)	0.307	0.94 (0.36–2.41)	0.889
Constipation	0.97 (0.46–1.98)	0.939	0.68 (0.23–1.85)	0.459
**Decreased platelet (< 50,000/mm**^**3**^**) with increased hematocrit (> 20%)**	**2.95 (1.18–7.40)**	**0.019**	**4.94 (1.48–17.31)**	**0.010**
Leucopenia	0.76 (0.39–1.46)	0.416	0.72 (0.28–1.83)	0.499

## Discussion

This was a large multicenter cross-sectional study of dengue among children, which reports the atypical and less reported clinical presentations, and models severity predictors of dengue in Bangladesh. ‘Mouth sore’ and ‘constipation’, two previously less reported gastrointestinal features, were prevalent among our participants. Atypical features were found mainly in the form of neurological manifestations, with confusion and blurring of vision being predominant. Age, mouth sore (in the form of sore throat and/or mouth ulcers), and decreased platelet with increased hematocrit were clinical predictors of severity.

Nearly one-third of cases had severe dengue in our study, which is higher than that (one-fifth of cases) found in 2018 by both Shultana et al. [27] and Afroze et al. [28] in two separate tertiary care hospitals (sample size, *n* = 89 and 106 respectively) in Dhaka city. Proportionately dengue shock syndrome cases were higher than any prior outbreaks recorded in the country [25, 27, 28]. This finding supports our hypothesis that the outbreak of 2019 was relatively more intense than the previous ones in terms of clinical severity. Besides, we found six cases of expanded dengue syndrome among whom at least two had features suggestive of encephalitis- a rare manifestation of dengue infection [34]. However, only 1.6% of children had a past history of dengue indicating that the severe cases cannot be explained by the secondary infection by a different serotype alone. The re-emergence of DEN-3 serotype in 2019 [15] combined with the fact that severe primary infection may occur in children [18] might explain the higher severity noted in this study. Moreover, several studies conducted within the last 5 years in India [35], Indonesia [36], and Mexico [37] found respectively 35, 20, and 58% severe dengue cases among their participants, supporting our hypothesis that dengue among children is occurring with increased severity.

Compared to the 2018 outbreak, children infected with dengue were older in the 2019 outbreak in the country [27, 28]. However, our age pattern matches the 2000 outbreak [25, 38], indicating a temporal variation in the age of involvement among children in this region. Additionally, we found that younger age is significantly associated with severe disease in multivariate analysis. This finding is also supported by surveillance studies from Asian countries [18].

A higher proportion of males among dengue-infected children of this study has been persistently noted in Bangladesh [27, 28] and several South-East Asian countries [32–35]. Anker and Arima [39] explored the sex-related differences in the prevalence of dengue in more detail. They noted that the magnitude of the difference is small and is not consistent in pediatric patients. However, male-female differences in the use of health services, the use of fully covered dresses by female children, and prioritizing provisions of male children in the society might be reasons for the differences noted in our country [40, 41].

Approximately three-fourths of `the families in this study came from the lower economic categories, with 33.7% having income less than 15,000 BDT per month (equivalent to USD 17.7). One explanation for this could be people with a lower monthly income are more likely to live in crowded places having a higher risk of contracting the disease from an infected person. Another possible reason is that public health facilities, from where our data was collected, are mostly visited by poor patients in the country [42].

The most frequent blood group among dengue infected children in our study was ‘O’ positive in contrast to the most common ABO and Rh blood type (‘B’ positive) noted among healthy donors of the country [43, 44]. Reports on the association between blood groups with dengue fever have been mixed. Khode et al. [45] found an association of ‘O’ positive blood groups with higher prevalence of dengue. But, in the study by Ravichandran et al. [46] it was ‘AB’ positive. Contrary to Kalayanarooj et al. [47] we did not find any association of severity of dengue with any particular blood ABO and Rh blood type. Therefore, the high prevalence of ‘O’ positive blood in our study could be a random finding.

Children with dengue show varying clinical presentations throughout the world. But fever is the most common symptom in dengue irrespective of types and severity. Variation exists in other symptoms and signs associated with fever. Likewise, we found a high-rise fever in all cases. Gastrointestinal features were more frequent in our study than that of previous year [48, 49]. Constipation, a seldom sought for feature in studies involving dengue [48, 49], was found to be more frequent than diarrhea in our patients. This might be consequent of decreased appetite and vomiting found in higher proportion in our study. However, the proportion of cases presenting with diarrhea was not less than other recent studies either [50, 51].

One unique finding of this study was independent association of sore mouth (including oral ulcers and/sore throat) with severe disease. Although, it was not found to be a factor for severity by Zhang and colleagues in their systematic review and meta-analysis of predictive symptoms and signs of severe dengue [52]. Sore mouth is a less common feature of dengue compared to other febrile illness which is usually reported as sore throat [51], therefore presence of it especially among younger children with dengue should raise concern regarding severity.

The most common bleeding manifestations was epistaxis and melena similar to several previous studies [53, 54]. A significantly higher proportion of melena was noted in severe dengue than the non-severe one consistent with previous evidence [25, 52].

The unusual features presented by children with dengue were mostly suggestive of nervous system involvement except palpitation and chest tightness which might have occurred due to hypovolemic shock associated cardiac ischemia [55] or due to transient involvement of cardiac muscles [32]. The neurological symptoms were suggestive of encephalopathy due to hypovolemic shock, and/or encephalitis and meningitis due to direct involvement of nervous tissue by the virus [56].

As older children could report their symptoms properly a higher proportion of pain related symptoms was noted among them. Interestingly female children also reported a higher frequency of different aches probably as a result of a lower threshold of pain sensitivity evident in women [57]. The high prevalence of mouth sores, rash, decreased appetite, and constipation among the youngest children might be explained by combinations of a developing immune system, decreased leucocyte counts, and a severe disease in this group.

We used conservative cut-off values for blood counts so that even slight elevation and reduction in specific investigations are not missed. Therefore, elevations in hematocrit value, and reductions in hemoglobin, leucocyte count, platelet count, were statistically similar between non-severe and severe dengue. But an elevation of hematocrit (> 20% from baseline) combined with a reduction in platelet count (< 50,000/mm^3^) was found to be a significant predictor of severe disease on multivariate analysis. Also, this was the only warning sign among others included in the multivariate logistic regression that came out as a significant predictor of dengue severity. Contrary to the estimates of Zhang et al. [52] our study did not find abdominal pain, vomiting and rash to be significant predictors of severity.

There were several limitations of our study. First, we had a small final sample size due to limited data collection and curation of incomplete data. Our data collection was limited in that we could not include the specialized centers dedicated for mother and child health in the city as because the data on pediatric dengue cases were collected in conjunction with adult cases from the multidisciplinary tertiary care hospitals. Second, we used a non-random convenience sampling, due to the rapidly evolving nature of the outbreak, to collect as many responses as possible near the end of peak time. This should be taken into consideration while interpreting our result. Third, the clinical classification of dengue was used for stratification of severity which might cause misclassification bias due to the subjective nature of the assessment. However, the initial classification of a patient during admission was subsequently verified by experts before discharge reducing the bias. Fourth, our design was cross-sectional in nature making the relations found between predictors and severity general associations.

## Conclusion

In 2019 outbreak, dengue among children was characterized by increased severity among preschoolers with a male predominance. Gastrointestinal symptoms were the most common presentation alongside fever. Epistaxis and melena were the main bleeding manifestations. Atypical manifestations were mostly suggestive of neurologic involvement. Decreasing age, presence of mouth sores and a decreased platelet with increased hematocrit were significant predictors of severity. Our findings would contribute to the clinical management and enrich knowledge in dengue research arena. Further randomized case-control and prospective studies with large samples are recommended to characterize particularities of pediatric dengue along with its classification, and complications..

## Supplementary Information

**Additional file 1.** Supplementary figures and tables.

**Additional file 2.** Questionnaire.

## Data Availability

The datasets used and/or analysed during the current study are available from the corresponding author on reasonable request.
