# Supplementary material for: Clinical spectrum and predictors of severity of dengue among children in 2019 outbreak: a multicenter hospital-based study in Bangladesh
Source: BMC Pediatr. 2021 Oct 29;21:478. doi: 10.1186/s12887-021-02947-y (PMC8555185; doi:10.1186/s12887-021-02947-y)
Supplement: Supplementary file 1 — Additional file 1. Supplementary figures and tables. [file 12887_2021_2947_MOESM1_ESM.docx]

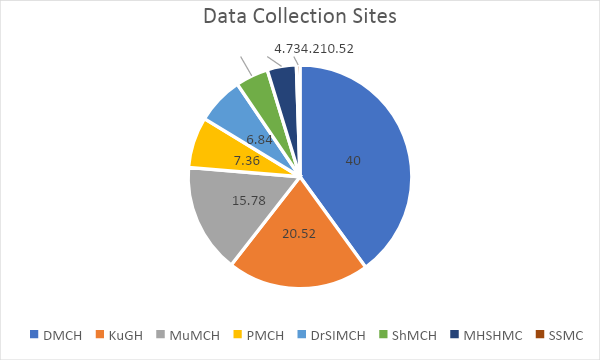


**Figure S1. Percentage of data collected from target hospitals**

(DMCH: Dhaka Medical College Hospital; KuGH: Kurmitola General Hospital; MuMCH: Mugda Medical College Hospital; PMCH: Popular Medical College Hospital; DrSIMCH: Dr Sirajul Islam Medical College Hospital; ShMCH: Suhrawardy Medical College Hospital; MHSHMC: MH Samorita Hospital and Medical College; SSMC: Sir Salimullah Medical College Hospital)

**Table S1. Defining features of dengue infection (adapted from national dengue management guideline 2018, Bangladesh^31^)**

| **Dengue type** | **Characteristics** |
| --- | --- |
| **Dengue fever (group A)** | **General features of fever without warning signs**    Live in/travel to dengue endemic area  Fever and two or more of the following criteria:  · Nausea, vomiting  · Rash  · Aches and pains  · Tourniquet test positive  · Leucopenia |
| **Dengue fever with warning signs (group B)** | **Criteria for group A plus one or more of warnings signs**    **Warning signs are:**  Abdominal pain or tenderness  Persistent vomiting  Clinical fluid accumulation  Mucosal bleed  Lethargy, restlessness  Liver enlargement >2cm  Laboratory: Increased in HCT concurrent with rapid decrease in platelet |
| **Severe dengue (group C)** | **Criteria for group B plus one or more of severe criteria**    **Severe criteria include:**  Severe plasma leakage leading to  · Shock  · Fluid accumulation with respiratory distress  Severe bleeding (as evaluated by clinician)  Severe organ involvement  · Liver enzymes: AST or ALT >1000  · CNS: Impaired consciousness  · Heart and other organ involvement |

**Table S2. Clinical features occurring in low frequency among participants by severity**

| **Variable** | **Non-severe**  **n (%)** | **Severe**  **n (%)** | **p** | **Total**  **n (%)** |
| --- | --- | --- | --- | --- |
| **Total** | **135 (71.1)** | **55 (28.9)** |  | **190 (100%)** |
| ***General manifestations*** |  |  |  |  |
| **Muco-cutaneous manifestations** |  |  |  |  |
| Pigmentation | 7 (5.4) | 5 (9.3) | 0.332 | 12 (6.5) |
| Hair loss | 9 (6.9) | 0 | 0.045 | 9 (4.9) |
| Skin Ulceration | 7 (5.3) | 2 (3.6) | 0.620 | 9 (4.8) |
| Bullous Dermatosis | 3 (2.3) | 0 | 0.258 | 3 (1.6) |
| **Joint manifestations** |  |  |  |  |
| Joint stiffness | 8 (19.5) | 0 | 0.111 | 8 (15.4) |
| Joint Swelling | 5 (11.4) | 0 | 0.187 | 5 (8.6) |
| Joint redness | 2 (4.7) | 0 | 0.487 | 2 (3.8) |
| **Hemorrhagic manifestation** |  |  |  |  |
| Sub-conjunctival hemorrhage | 4 (3.0) | 4 (7.3) | 0.180 | 8 (4.2) |
| Gum bleeding | 7 (5.2) | 0 | 0.085 | 7 (3.7) |
| Hematemesis | 2 (1.5) | 3 (5.5) | 0.121 | 5 (2.6) |
| Hematuria | 2 (1.5) | 2 (3.6) | 0.348 | 4 (2.1) |
| Menorrhagia | 2 (1.5) | 2 (3.6) | 0.348 | 4 (2.1) |
| Hemoptysis | 1 (0.7) | 2 (3.6) | 0.146 | 3 (1.6) |
| Ecchymosis | 1 (0.7) | 1 (1.8) | 0.509 | 2 (1.1) |
| ***Unusual manifestations*** |  |  |  |  |
| **Neurological symptoms** |  |  |  |  |
| Sensory impairment | 12 (9.2) | 6 (11.1) | 0.684 | 18 (9.7) |
| Convulsion | 5 (3.8) | 8 (14.8) | 0.007 | 13 (7.0) |
| Disorientation | 7 (5.3) | 4 (7.3) | 0.602 | 11 (5.9) |
| Neck stiffness | 7 (5.2) | 4 (7.7) | 0.522 | 11 (5.9) |
| Auditory Hallucination | 2 (1.5) | 3 (5.6) | 0.120 | 5 (2.7) |
| Transient Amnesia | 3 (2.3) | 0 | 0.264 | 3 (1.6) |
| Altered mental state | 2 (1.5) | 1 (1.9) | 0.864 | 3 (1.6) |
| Facial deviation | 0 | 1 (1.9) | 0.116 | 1 (0.5) |
| **Others** |  |  |  |  |
| Chest tightness | 13 (10.0) | 5 (9.4) | 0.907 | 18 (9.8) |
| Palpable lymph nodes | 2 (1.8) | 3 (6.4) | 0.127 | 5 (3.1) |
| ***Signs*** |  |  |  |  |
| Cyanosis | 1 (0.8) | 3 (5.6) | 0.042 | 4 (2.2) |
| Hepatomegaly | 0 | 3 (6.1) | 0.007 | 3 (1.8) |
| Jaundice | 0 | 1 (1.9) | 0.122 | 1 (0.6) |
| **Features of plasma leakage and shock** |  |  |  |  |
| Ascites | 6 (4.4) | 7 (12.7) | 0.04 | 13 (6.8) |
| Pleural effusion | 3 (2.2) | 8 (14.5) | 0.001 | 11 (5.8) |
| Anasarca | 1 (0.7) | 4 (7.3) | 0.011 | 5 (2.6) |
| Leg edema | 0 | 1 (1.8) | 0.116 | 1 (0.5) |

Data is presented as mean±SD and n (%) as appropriate; Percentage is expressed among available responses (after excluding missing values)

p value determined by chi-square test and independent samples t test

**Table S3. Clinical and Investigation profile of patients in relation to sex (n=189)**

| **Variable** | **Female**  **n(%)** | **Male**  **n(%)** | **P value** |
| --- | --- | --- | --- |
| Total N (%) | 86 (45.5) | 103 (54.5) |  |
| **Fever and associated features** |  |  |  |
| Fever | 86 (100) | 103 (100) | NA |
| Highest recorded temperature | 103.3±1.3 | 103.3±1.2 | 0.868 |
| Duration of fever (days) | 5.2±1.8 | 5.5±3.4 | 0.910 |
| Lethargy | 64 (77.1) | 76 (79.2) | 0.739 |
| Headache | 59 (70.2) | 67 (66.3) | 0.571 |
| Backache | 36 (45.0) | 30 (30.3) | 0.043 |
| Retroorbital pain | 35 (43.2) | 28 (27.7) | 0.029 |
| **Muco-cutaneous manifestations** |  |  |  |
| Itching | 35 (41.2) | 27 (26.7) | 0.037 |
| Mouth sores | 25 (29.4) | 27 (26.7) | 0.685 |
| Rash | 24 (28.6) | 28 (27.2) | 0.833 |
| Pigmentation | 6 (7.2) | 6 (6.0) | 0.738 |
| Hair loss | 8 (9.4) | 1 (1.0) | 0.008 |
| Skin ulceration | 2 (2.4) | 7 (6.9) | 0.152 |
| Bullous dermatosis | 2 (2.4) | 1 (1.0) | 0.456 |
| **Joint manifestations** |  |  |  |
| Arthralgia | 40 (48.2) | 34 (34.0) | 0.051 |
| Joint swelling | 4 (13.8) | 1 (3.4) | 0.160 |
| Joint redness | 2 (7.7) | 0 | 0.142 |
| Joint stiffness | 4 (15.4) | 4 (15.4) | 1.000 |
| ***Danger ‘signs’*** |  |  |  |
| **Gastrointestinal features** |  |  |  |
| Vomiting | 72 (84.7) | 79 (76.7) | 0.169 |
| Decreased appetite | 73 (84.9) | 77 (74.8) | 0.087 |
| Abdominal Pain | 62 (72.1) | 59 (58.4) | 0.051 |
| Loose motion | 41 (52.3) | 39 (38.2) | 0.192 |
| Constipation | 19 (23.8) | 31 (30.4) | 0.319 |
| **Hemorrhagic manifestation** |  |  |  |
| Any Hemorrhage | 23 (29.5) | 18 (18.4) | 0.083 |
| Nasal bleeding (Epistaxis) | 5 (5.8) | 7 (6.8) | 0.783 |
| Malena | 4 (4.7) | 7 (6.8) | 0.531 |
| Sub-conjunctival hemorrhage | 5 (5.8) | 3 (2.9) | 0.324 |
| Gum bleeding | 5 (5.8) | 2 (1.9) | 0.160 |
| Hematemesis | 2 (2.3) | 3 (2.9) | 0.802 |
| Hematuria | 3 (3.5) | 1 (1.0) | 0.231 |
| Menorrhagia | 4 (4.7) | 0 | 0.027 |
| Hemoptysis | 1 (1.2) | 2 (1.9) | 0.670 |
| Ecchymosis | 1 (1.2) | 1 (1.0) | 0.898 |
| ***Unusual manifestations*** |  |  |  |
| **Neurological manifestations** |  |  |  |
| Confusion | 20 (23.3) | 20 (20.0) | 0.590 |
| Blurring of vision | 13 (15.3) | 15 (14.7) | 0.911 |
| Sensory impairment | 9 (10.7) | 9 (9.0) | 0.697 |
| Convulsion | 3 (3.5) | 10 (10.0) | 0.082 |
| Disorientation | 6 (7.0) | 5 (5.0) | 0.569 |
| Neck stiffness | 2 (2.3) | 9 (9.1) | 0.052 |
| Auditory Hallucination | 0 | 5 (5.0) | 0.038 |
| Transient Amnesia | 1 (1.2) | 2 (2.0) | 0.672 |
| Altered mental state | 1 (1.2) | 2 (2.0) | 0.665 |
| Facial deviation | 1 (1.02) | 0 | 0.274 |
| **Others** |  |  |  |
| Palpitation | 11 (13.1) | 11 (11.0) | 0.663 |
| Chest tightness | 8 (9.5) | 10 (10.2) | 0.878 |
| Palpable lymph nodes | 2 (2.7) | 3 (3.5) | 0.766 |
| ***Signs*** |  |  |  |
| Anemia/Pallor | 15 (18.8) | 12 (12.2) | 0.229 |
| Dehydration | 11 (13.9) | 10 (10.4) | 0.477 |
| Cyanosis | 0 | 4 (4.0) | 0.067 |
| Hepatomegaly | 1 (1.3) | 2 (2.2) | 0.677 |
| Jaundice | 1 (1.2) | 0 | 0.267 |
| **Features of plasma leakage and shock** |  |  |  |
| Clinical accumulation of fluid | 14 (17.3) | 15 (14.9) | 0.656 |
| Cold clammy skin | 40 (48.2) | 46 (46.5) | 0.816 |
| Excessive sweating | 38 (45.8) | 37 (37.0) | 0.229 |
| Oliguria/Anuria | 26 (31.7) | 22 (22.0) | 0.139 |
| Dyspnoea | 13 (15.3) | 17 (16.8) | 0.776 |
| Loss of/impaired consciousness | 5 (6.0) | 8 (8.2) | 0.566 |
| Ascites | 4 (4.7) | 9 (8.7) | 0.269 |
| Pleural effusion | 6 (7.0) | 5 (4.9) | 0.535 |
| Anasarca | 3 (3.5) | 2 (1.9) | 0.509 |
| Leg edema | 0 | 1 (1.0) | 0.360 |
| ***Investigation profile*** |  |  |  |
| **Hematological manifestations** |  |  |  |
| Increased hematocrit (>20% from baseline) | 13 (15.5) | 12 (11.9) | 0.461 |
| Reduced hemoglobin level (<age adjusted minimum) | 34 (40.0) | 37 (36.3) | 0.601 |
| Thrombocytopenia (<150000/mm^3^) | 77 (89.5) | 86 (85.1) | 0.371 |
| Leucopenia (<4000/ mm^3^) | 44 (54.3) | 28 (29.2) | 0.001 |
| **Dengue classification by symptom** |  |  |  |
| DF | 52 (60.5) | 69 (67.0) | 0.029 |
| DHF | 9 (10.5) | 10 (9.7) |  |
| DSS | 25 (29.1) | 18 (17.5) |  |
| EDS | 0 | 6 (5.8) |  |
| **Dengue classification by severity** |  |  |  |
| Group A (Dengue) | 15 (17.4) | 24 (23.3) | 0.432 |
| Group B (Dengue with warning signs) | 43 (50.0) | 53 (51.5) |  |
| Group C (Severe dengue) | 28 (32.6) | 26 (25.2) |  |

Data is presented as mean±SD and n (%) where appropriate; Percentage is expressed among available responses (after excluding missing values);

p value determined by chi-square test and independent samples t test where appropriate;

ALT: Alanine transaminase; AST: Aspartate transaminase; PT: Prothrombin time; APTT: Activated Partial Thromboplastin Time; DF: Dengue Fever; DHF: Dengue Hemorrhagic fever; DSS: Dengue Shock Syndrome; EDS: Expanded Dengue Syndrome

**Table S4. Clinical and investigation profile of patients in relation to age (n=190)**

| **Variables** | **<5 years**  **n (%)** | **5-9 years**  **n (%)** | **10-14 years**  **n (%)** | **P-value** |
| --- | --- | --- | --- | --- |
| Total N (%) | 27 (14.2) | 77 (40.5) | 86 (45.3) |  |
| **Fever and associated features** |  |  |  |  |
| Fever | 27 (100) | 77 (100) | 86 (100) | NA |
| Highest recorded temperature | 102.6±1.4 | 103.5±1.1 | 103.3±1.2 | 0.022 |
| Duration of fever | 5.0±2.0 | 5.9±3.6 | 5.0±2.1 | 0.214 |
| Lethargy | 16 (64.0) | 63 (82.9) | 62 (78.5) | 0.138 |
| Headache | 11 (47.8) | 45 (58.4) | 70 (81.4) | 0.001 |
| Backache | 9 (39.1) | 18 (24.0) | 39 (47.6) | 0.009 |
| Retroorbital pain | 8 (34.8) | 23 (30.3) | 32 (38.1) | 0.581 |
| **Muco-cutaneous manifestations** |  |  |  |  |
| Itching | 6 (23.1) | 29 (37.7) | 27 (33.2) | 0.794 |
| Mouth sores | 13 (38.2) | 24 (31.2) | 17 (20.2) | 0.029 |
| Rash | 13 (48.1) | 22 (29.3) | 17 (19.8) | 0.015 |
| Pigmentation | 2 (7.7) | 7 (9.3) | 3 (3.6) | 0.336 |
| Hair loss | 0 (0.0) | 2 (2.7) | 7 (8.3) | 0.117 |
| Skin ulceration | 0 (0.0) | 7 (9.2) | 2 (2.4) | 0.061 |
| Bullous dermatosis | 2 (7.7) | 1 (1.3) | 0 | 0.024 |
| **Joint manifestations** |  |  |  |  |
| Arthralgia | 8 (33.3) | 35 (46.7) | 32 (37.6) | 0.373 |
| Joint stiffness | 1 (20.0) | 2 (8.7) | 5 (20.8) | 0.492 |
| Joint swelling | 0 | 3 (12.0) | 2 (7.4) | 0.613 |
| Joint redness | 0 | 0 | 2 (7.7) | 0.340 |
| ***Danger ‘signs’*** |  |  |  |  |
| **Gastrointestinal features** |  |  |  |  |
| Vomiting | 17 (63.0) | 66 (86.8) | 69 (80.2) | 0.027 |
| Decreased appetite | 10 (37.0) | 10 (13.0) | 19 (22.1) | 0.026 |
| Abdominal Pain | 12 (46.2) | 49 (63.6) | 61 (71.8) | 0.054 |
| Loose motion | 5 (18.5) | 30 (39.5) | 46 (53.5) | 0.004 |
| Constipation | 13 (48.1) | 13 (31.1) | 14 (17.1) | 0.005 |
| **Hemorrhagic manifestations** |  |  |  |  |
| Any Hemorrhage | 3 (11.1) | 24 (35.8) | 15 (18.1) | 0.010 |
| Nasal bleeding | 2 (7.4) | 6 (7.8) | 4 (4.7) | 0.690 |
| Malena | 1 (3.7) | 8 (10.4) | 2 (2.3) | 0.078 |
| Sub-conjunctival hemorrhage | 0 | 6 (7.8) | 2 (2.3) | 0.111 |
| Gum bleeding | 0 | 4 (5.2) | 3 (3.5) | 0.464 |
| Hematemesis | 0 | 4 (5.2) | 1 (1.2) | 0.180 |
| Hematuria | 0 | 1 (1.3) | 3 (3.5) | 0.444 |
| Menorrhagia | 0 | 0 | 4 (4.7) | 0.085 |
| Hemoptysis | 0 | 3 (3.9) | 0 | 0.107 |
| Ecchymosis | 0 | 2 (2.6) | 0 | 0.227 |
| ***Unusual manifestations*** |  |  |  |  |
| **Neurological manifestations** |  |  |  |  |
| Confusion | 2 (7.7) | 20 (26.0) | 18 (21.4) | 0.145 |
| Blurring of vision | 1 (3.8) | 13 (16.9) | 14 (16.5) | 0.233 |
| Sensory impairment | 0 | 9 (11.8) | 9 (10.6) | 0.218 |
| Convulsion | 3 (11.5) | 8 (10.4) | 2 (2.4) | 0.084 |
| Disorientation | 1 (3.8) | 5 (6.5) | 5 (6.0) | 0.884 |
| Neck stiffness | 1 (3.8) | 5 (6.6) | 5 (6.0) | 0.878 |
| Auditory Hallucination | 1 (4.0) | 1 (1.3) | 3 (3.5) | 0.616 |
| Transient Amnesia | 0 | 1 (1.3) | 2 (2.4) | 0.680 |
| Altered mental state | 0 | 2 (2.6) | 1 (1.2) | 0.610 |
| Facial deviation | 0 | 1 (1.3) | 0 | 0.488 |
| **Others** |  |  |  |  |
| Palpitation | 1 (4.2) | 6 (7.9) | 15 (17.9) | 0.074 |
| Chest tightness | 1 (4.2) | 5 (6.6) | 12 (14.5) | 0.151 |
| Palpable lymph nodes | 2 (10.0) | 0 | 3 (3.8) | 0.072 |
| ***Signs*** |  |  |  |  |
| Anemia/Pallor | 3 (11.1) | 7 (10.1) | 17 (20.5) | 0.171 |
| Dehydration | 4 (16.0) | 4 (5.8) | 13 (15.9) | 0.131 |
| Cyanosis | 1 (3.8) | 1 (1.3) | 2 (2.4) | 0.730 |
| Jaundice | 0 | 0 | 1 (1.2) | 0.559 |
| Hepatomegaly | 0 | 2 (3.0) | 1 (1.2) | 0.580 |
| **Features of plasma leakage e and shock** |  |  |  |  |
| Clinical accumulation of fluid | 6 (22.2) | 15 (20.8) | 8 (9.5) | 0.096 |
| Cold clammy skin | 9 (34.6) | 45 (60.0) | 33 (40.2) | 0.017 |
| Excessive sweating | 9 (34.6) | 33 (44.0) | 33 (39.8) | 0.681 |
| Oliguria/Anuria | 9 (34.6) | 24 (32.4) | 15 (18.1) | 0.072 |
| Dyspnea | 3 (11.5) | 10 (13.2) | 18 (21.2) | 0.298 |
| Loss of/impaired consciousness | 2 (8.0) | 8 (10.7) | 4 (4.9) | 0.408 |
| Ascites | 3 (11.1) | 7 (9.1) | 3 (3.5) | 0.235 |
| Pleural effusion | 1 (3.7) | 6 (7.8) | 4 (4.7) | 0.611 |
| Anasarca | 2 (7.4) | 1 (1.3) | 2 (2.3) | 0.227 |
| Leg oedema | 0 | 1 (1.3) | 0 | 0.478 |
| ***Investigations*** |  |  |  |  |
| **Hematological profile** |  |  |  |  |
| Increased hematocrit (>20% from baseline) | 3 (11.5) | 11 (14.7) | 11 (12.9) | 0.324 |
| Reduced hemoglobin level (<age adjusted minimum) | 15 (57.7) | 28 (36.8) | 29 (33.7) | 0.083 |
| Thrombocytopenia (<150000/ mm^3^) | 18 (69.2) | 67 (87.0) | 79 (92.9) | 0.007 |
| Leucopenia (<4000/ mm^3^) | 7 (29.2) | 33 (45.8) | 32 (39.0) | 0.332 |
| **Dengue classification by symptom** |  |  |  |  |
| DF | 14 (51.9) | 42 (54.5) | 65 (75.6) | <0.001 |
| DHF | 4 (14.8) | 7 (9.1) | 19 (10.0) |  |
| DSS | 5 (18.5) | 27 (35.1) | 12 (14.0) |  |
| EDS | 4 (14.8) | 1 (1.3) | 1 (1.2) |  |
| **Dengue classification by severity** |  |  |  |  |
| Group A (Dengue) | 3 (11.1) | 3 (3.9) | 33 (38.4) | <0.001 |
| Group B (Dengue with warning signs) | 13 (48.1) | 45 (58.4) | 38 (44.2) |  |
| Group C (Severe dengue) | 11 (40.7) | 29 (37.7) | 15 (17.4) |  |

Data is presented as mean±SD and n (%) as appropriate; Percentage is expressed among available responses (after excluding missing values);

p value determined by chi-square test and independent samples t test as appropriate;

ALT: Alanine transaminase; AST: Aspartate transaminase; PT: Prothrombin time; APTT: Activated Partial Thromboplastin Time; DF: Dengue Fever; DHF: Dengue Hemorrhagic fever; DSS: Dengue Shock Syndrome; EDS: Expanded Dengue Syndrome
